# Detours to Replication: Functions of Specialized DNA Polymerases during Oncogene-induced Replication Stress

**DOI:** 10.3390/ijms19103255

**Published:** 2018-10-20

**Authors:** Wei-Chung Tsao, Kristin A. Eckert

**Affiliations:** Department of Pathology, The Jake Gittlen Laboratories for Cancer Research, Hershey, PA 17033, USA; wxt139@psu.edu

**Keywords:** Difficult-to-Replicate Sequences, replication stress, non-B DNA, Polymerase eta, Polymerase kappa, genome instability, common fragile sites, Microsatellites

## Abstract

Incomplete and low-fidelity genome duplication contribute to genomic instability and cancer development. Difficult-to-Replicate Sequences, or DiToRS, are natural impediments in the genome that require specialized DNA polymerases and repair pathways to complete and maintain faithful DNA synthesis. DiToRS include non B-DNA secondary structures formed by repetitive sequences, for example within chromosomal fragile sites and telomeres, which inhibit DNA replication under endogenous stress conditions. Oncogene activation alters DNA replication dynamics and creates oncogenic replication stress, resulting in persistent activation of the DNA damage and replication stress responses, cell cycle arrest, and cell death. The response to oncogenic replication stress is highly complex and must be tightly regulated to prevent mutations and tumorigenesis. In this review, we summarize types of known DiToRS and the experimental evidence supporting replication inhibition, with a focus on the specialized DNA polymerases utilized to cope with these obstacles. In addition, we discuss different causes of oncogenic replication stress and its impact on DiToRS stability. We highlight recent findings regarding the regulation of DNA polymerases during oncogenic replication stress and the implications for cancer development.

## 1. Introduction

To maintain genome integrity, complete genome duplication requires careful orchestration of the replication machinery through active coordination of DNA synthesis and repair. Encounters with structural impediments in the genome can lead to the slowing or stalling of the replication fork. This phenomenon, termed replication stress, results in uncoupling of the helicase from the replisome polymerases, creating long stretches of single-stranded DNA and activating a cascade of signaling pathways referred to here as the replication stress response [1]. DNA replication stress has emerged as a key factor driving genome instability during tumor cell evolution [2,3,4]. Persistent replicative stress and unresolved fork stalling leads to repair processing and/or collapse of stalled DNA replication forks, ultimately resulting in double strand breaks [3,5]. The replication stress response coordinates DNA replication initiation and elongation, with the ability to rescue and resume synthesis at stalled replication forks [1,5].

Replicative polymerases encounter many Difficult-To-Replicate Sequences, or DiToRS, regions of the genome that hinder DNA synthesis elongation [6,7,8]. Impediments to genome replication arise from both endogenous and exogenous sources. Endogenous replication fork barriers include naturally arising physical obstacles, such as protein-DNA complexes or transcription-replication collisions (e.g., R-loops), and non-B DNA secondary structures, such as those formed within microsatellites, common fragile sites (CFSs), and telomeres [5,9,10,11,12,13]. Here, we will focus on DiToRS formed by non B-DNA secondary structures and repetitive DNA. For a detailed review of physical obstacles from DiToRS, we recommend these sources [7,14]. Exogenous exposures that impede DNA replication include DNA lesions formed by environmental and physical insults, such as carcinogenic chemicals (i.e. tobacco smoke), chemotherapeutic agents, or irradiation [5].

DiToRS present a challenge to DNA synthesis by replicative DNA polymerases and thereby contribute to endogenous replication stress. The bulk of eukaryotic genome duplication is carried out by the B-family replicative polymerases δ and ε. These polymerases are highly processive enzymes with proofreading domains that maintain faithful DNA synthesis. However, the biochemical nature of the replicative DNA polymerases renders the catalytic site inefficient at synthesizing past DiToRS and DNA lesions. For this, cells utilize specialized DNA polymerases that have increased flexibility for substrates, allowing enzymatic bypass of unusual DNA structures [15]. Importantly, decades of research have revealed the importance of specialized DNA polymerases in mediating DNA synthesis past DiToRS as well as DNA lesions to prevent replication stress.

Oncogene activation is a hallmark of cancer and typically exerts a myriad of effects on cellular processes including, but not limited to, metabolism, proliferation, cell cycle progression, transcription and DNA replication. Of note, oncogenes can disrupt replication dynamics by altering origin licensing, origin firing, deoxynucleotide pools and transcription, leading to persistent replicative stress and genome instability. Additionally, hyper-replication causes increased chromosomal breakage at DiToRS such as CFSs [3,4,16], theoretically placing a reliance on specialized DNA polymerases to complete genome duplication and cell proliferation. In this review, we summarize the types of DiToRS with a focus on sequences that adopt non-B DNA structures and the importance of specialized polymerases for completing DiToRS replication. Furthermore, we discuss how oncogenic replication stress affects the regulation of specialized DNA polymerases during oncogenic replication stress, and the implications for carcinogenesis.

## 2. Endogenous Genome DiToRS

The human genome is characterized by its DNA sequence complexity and high repetitive DNA content [17]. With distinct sequence properties such as base composition, symmetry, and length, repetitive sequences can form DNA secondary structures alternative to the right-handed B-DNA helix [18] more favorably than random DNA sequences (reviewed in [7,11]). For instance, left-handed Z-DNA duplexes form within alternating purine-pyrimidine sequences [19]; H-DNA triplexes form within polypurine/polypyrimidine tracts and mirror repeats [20,21]; and both intramolecular and intermolecular four stranded structures form within repeats rich in adjacent guanines [22] such as telomeric repeats [23]. In addition, specific motifs can form unique, localized structures, including, but not limited to, bent DNA within A-rich tracts [24], G quartets or G quadruplex (G4) structures formed within G-rich tracts [25], and cruciform structures and hairpins formed between inverted repeats or quasipalindromes [26,27].

The presence of repetitive sequences is a major factor impacting genome stability, and non-B DNA secondary structures play an important, causative role in human cell mutagenesis and disease [28,29,30]. DiToRS exert their effects on genome instability by interfering with DNA synthesis accompanying any phase of DNA metabolism: replication, repair, or recombination. Below, we summarize evidence for the effect of DiToRS on replication obtained through *in vitro* studies of purified polymerases, *ex vivo* studies using reporter plasmids and two-dimensional (2D) gel analyses of fork progression, and *in vivo* studies analyzing replication progression within individual DNA molecules. Such DiToRS are linked mechanistically to genome variations that underlie inherited microsatellite expansion diseases [31], *de novo* genomic disorders [32,33], cancer genome instability [34,35,36], and genome evolution [37]. Evolutionarily, conserved repetitive elements prone to breakage or viral integration provide ideal regions for chromosomal rearrangement and species divergence [38].

DiToRS best described at the nucleotide level as inhibiting replication are associated with microsatellites and chromosomal fragile sites. Microsatellites are short tandem repeats of 1–6 basepairs per unit that are distributed throughout the human genome in both inter- and intragenic regions [39,40]. As detailed below, many microsatellite sequences can adopt alternative secondary structures, the form and stability being dependent on the repeat unit sequence composition and total allele length. Fragile sites are specific chromosomal regions where a high frequency of gaps/breaks can be observed in metaphase chromosomes [41], and include CFS and rare fragile sites [42,43], early replicating fragile sites [10], and telomeres [12]. CFS regions are associated with recurrent translocations, interstitial deletions, and amplifications in cancer genomes [44,45], copy number variation in stem cells [46], and viral DNA integration events [47,48]. A vast literature supports a role for DiToRS as contributing to CFS etiology, and breakage within CFS regions is enhanced by replication stress (reviewed in [8,41,49,50]). However, additional genome features and mechanisms contribute to difficult replication through CFS regions, including a paucity of replication origins [51], inefficient replication initiation [52,53], and the formation of R loops during transcription and collision with replication forks during S phase [54].

### 2.1. AT-Rich Repeats

CFSs are enriched in *Alu* repeats and contain highly AT-rich regions, particularly mononucleotide [A/T] microsatellites [2,55]. Such AT-rich, high DNA “flexibility regions” may affect replication by hindering efficient topoisomerase activity ahead of the replication fork [42,56]. The Flex 1 region of FRA16D contains a [AT/TA]_34_ microsatellite that induces replication fork stalling and chromosomal fragility in an *S. cerevisiae* model [13]. Using locus-specific fiber analyses and FANCD2-deficient human cells, replication forks were shown to stall within the AT-rich flexibility core regions of FRA16D [57]. Similarly, DNA fiber analyses demonstrated that replication through the FRA16C locus was slowed near AT-rich regions [52]. The rare fragile site FRA16B spans the same genomic locus as FRA16C, but is an expanded, AT-rich minisatellite repeat. *In vitro*, 14 copies of the 33mer minisatellite repeat were shown to form alternative DNA secondary structures and when present in reporter plasmids, inhibited replication in human cells [58].

Our laboratory provided direct experimental evidence that specific DiToRS within CFSs, namely [A/T] and [AT/TA] microsatellites, are inhibitory to human replicative DNA polymerases. A mononucleotide [A/T] repeat of 28 units within the FRA16D Flex 5 region inhibited DNA synthesis *in vitro* by the replicative polymerases α-primase and δ, and inhibit DNA synthesis in cell-free human extracts [59]. The human Pol δ holoenzyme dissociates from the DNA template at such repeat elements [60], which may contribute to impaired replication fork progression observed within FRA16D. Polymerase pausing may be due to the formation of bent DNA within the [A/T] tract [61], rather than H-DNA formation [59]. Hairpin structures formed within long, CFS-derived [AT] repeats (25 units or greater) also impede Pol δ holoenzyme synthesis [62], consistent with the length dependence of replication inhibition and chromosomal instability at [AT/TA] tracts observed *in vivo* [13]. Interestingly, a genome-wide analysis of structural variation in cancer genomes found a significant enrichment of [AT/TA] repeats at translocation endpoints, whereas [A/T] repeats were found preferentially at deletion endpoints [34].

### 2.2. GC-Rich Repeats

Arguably the best studied DiToRS in the human genome are those formed within expanded microsatellites associated with over 30 neurological and neuromuscular disorders. The types of DiToRS formed within these repetitive sequences and their effects on DNA metabolism have been recently reviewed [31,63]. The [CCG/CGG] repeats can form both hairpins and G4 structures. Early studies from the Usdin lab showed that [CGG] and other G-rich sequences are barriers to *in vitro* DNA synthesis by prokaryotic polymerases, consistent with formation of intrastrand quadruplex structures [64,65]. Using reporter plasmids, these repeats were shown by 2D gel analysis to stall replication in a length-dependent manner, in both yeast and primate cells [66,67]. Telomeric sequences also encode GC-rich repeats that can fold into G4 structures [68]. Pol δ is the major DNA polymerase responsible for human telomere ([TTAGGG] repeat) synthesis [69]. However, the Opresko lab showed that while Pol δ pauses during synthesis of telomeric repeats *in vitro*, this pausing is not the result of G4 structure formation [70]. Never-the-less, predicted G4-motifs are enriched at the breakpoints of somatic copy number variations found in human cancers [71].

### 2.3. Triplex DNA (H-DNA)

Naturally occurring H DNA-sequences are a source of double strand breaks and genome instability [36,72], and sequences with H-DNA potential, particularly [GAA] and [GAAA] microsatellites, are associated with translocation breakpoints in tumor cells [34,36]. Our lab has shown that the formation of H-DNA during long [TC] microsatellite DNA synthesis *in vitro* inhibits replicative Pol α-primase [73]. However, the best studied example of an H-DNA forming DiToRS is the expanded [GAA/CTT] repeat causing Freidrich’s ataxia. Using plasmid reporter assays and 2D gel analyses, replication pausing within the repeats was observed in yeast, mammalian, and human cell systems [74,75,76]. Direct visualization of replication fork intermediates using electron microscopy confirmed the presence of aberrant structures within the long [GAA/CTT] repeats [76].

### 2.4. Inverted Repeats and Quasipalindromes

Inverted repeats and quasipalindromes are hot spots of double-strand breaks and rearrangements that contribute to genomic instability [77,78]. Palindromes formed by *Alu* elements (long inverted repeats) cause replication stalling in vivo [79]. Our lab demonstrated that much shorter, quasipalindrome repeats (from 29–37 nucleotides in length) found within CFSs can directly impede lagging strand polymerases *in vitro* [59,60]. Short inverted repeats (<30 bp) are enriched at cancer genome translocation breakpoints, and a short inverted repeat present in a reporter plasmid was sufficient to impede DNA replication fork progression in primate cells [80].

## 3. Specialized DNA Polymerases and the Maintenance of DiToRS Stability

Of the 15 human DNA polymerases, Pol zeta (Pol ζ) from the B-family, and Pols eta (Pol η), kappa (Pol κ), and Rev1 from the Y-family are known regulators of DiToRS stability. These polymerases are best known for their ability to carry out bypass of specific DNA lesions that block replicative polymerases, hence their common description as translesion synthesis (TLS) polymerases [81]. We proposed the terminology “specialized polymerases” as a more general term than “TLS polymerases”, given the known cellular roles of these same enzymes in DiToRS replication [82]. For detailed reviews of the replicative and specialized DNA polymerases required for DNA repair, including TLS, homologous recombination, and non-homologous end joining, see Sale, 2013 [83]; Barnes, 2017 [84]; Bournique, 2018 [85]; Vaisman, 2017 [15]. Specialized polymerases are generally considered to be error-prone because of their low fidelity compared to replicative polymerases when copying an undamaged, B-form DNA templates. However, when utilizing templates containing DNA lesions or non-B DNA structures, these polymerases can replicate DNA with remarkable accuracy and efficiency [86].

**Figure 1:**
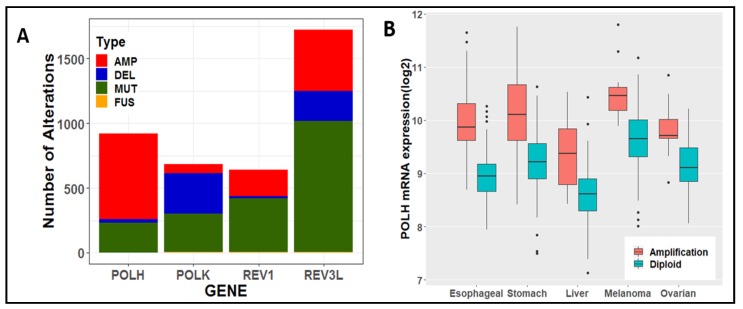
Specialized DNA Polymerase Gene Alterations in Cancer. Thirty tumor groups (*n* = 65,690 samples) were queried using cBioportal (www.cbioportal.org) and public datasets available as of 1 September 2018. (**A**) The total number of *POLH, POLK, REV3L, and REV1* gene alterations found in all tumor types are categorized into mutations (green bars), amplifications (red bars), deep deletions (blue bars), fusions (orange bars). Fusion events found in tumors were extremely rare: *POLH* has 2; *POLK* has 3; *REV1* has 3; *REV3L* has 5. Multiple alterations with mutations were classified as mutations. Multiple alterations with fusions only occurred with amplifications and thus were designated as amplifications. (**B**) Correlation of *POLH* gene expression with copy number status. Individual TCGA PanCancer Atlas datasets for each type of cancer with mRNA expression from RNA-seq data sets were extracted as RSEM and graphed as box-and-whisker plots.

Given their known functions in maintaining genome stability, surprisingly little is known about altered expression or mutation of specialized DNA polymerase genes in tumors. Using cBioPortal analyses [87,88], we observed that *POLH* (Pol η), *POLK* (Pol κ), *REV3L* (Pol ζ) and *REV*1 genes display different types of genomic alterations (Figure 1A). In total, an average of 6% of all tumor samples queried have variant specialized polymerase genes, although alterations within certain types of cancer reach up to 18%. The *POLH* locus is primarily amplified in cancers, and this amplification is correlated with increased mRNA expression (Figure 1B). Increased *POLH* expression has also been reported in Non-Small Cell Lung Cancers [89] and Head and Neck Squamous Cell Cancers [90]. Inherited loss-of-function *POLH* mutations cause Xeroderma Pigmentosum Variant (XPV), a disease characterized by skin UV hypersensitivity and predisposition to skin cancer [91,92]. Correspondingly, the most studied biochemical activity of Pol η is its ability to accurately replicate UV-induced cyclobutane pyrimidine dimers and other lesions [92,93]. Structurally, Pol η has a unique little finger domain that may act as a molecular splint by forcing the DNA to adopt a B-DNA form during DNA synthesis [94]. Loss of Pol η results in increased mutagenesis induced by UV and other DNA damaging agents [95,96], and increases genome instability at CFSs (see below). Furthermore, Pol η has roles in additional cellular processes, including mismatch repair [97], homologous recombination [98], and somatic hypermutation [99]. A putative Pol η signature has been found in several cancers, including melanoma and esophageal cancer [100], both of which are amplified in our analyses (Figure 1B). Thus, Pol η’s role in tumorigenesis is more complex than once thought, and could be either tumor suppressive or oncogenic, depending on the cellular context.

In contrast to *POLH*, the *POLK* locus is highly deleted in cancers. Decreased *POLK* expression has been noted in several studies, including ovarian, stomach, lung, and colorectal cancers [101,102,103]. Biochemically, Pol κ specializes in error-free bypass of bulky minor groove N2-deoxyguanine lesions, such as benzo(a)pyrene diolepoxide (BPDE) adducts. This ability is due, in part, to its unique N-clasp domain that allows the enzyme to encircle the DNA while accommodating bulkier lesions in the closed conformation [104]. Pol κ also plays a role in DNA repair processes such as nucleotide excision repair [105], double-stranded break repair [106], and induction of replication stress signaling via ATR (see below). Pol κ-deficient cells have elevated levels of BPDE-induced mutagenesis [107] and enhanced ATR checkpoint signaling [108]. However, the presence of Pol κ is also a source of mutagenesis due to its low fidelity on undamaged, non-repetitive DNA templates [109], and Pol κ overexpression increases DNA damage foci and homologous recombination [110]. Thus, Pol κ must be tightly regulated during cellular replication and repair processes to maintain genome stability.

Roles for *REV1* and *REV3* in lung cancer [111] have also been documented, and were among the most altered tumor samples in our analysis. Rev1 is a deoxycytidyl transferase that is restricted to inserting dCTPs opposite guanines and abasic sites [112,113]. Rev1 incorporates dCTPs by evicting the template guanine and instead relying on an arginine residue in the catalytic site to bind incoming dCTPs [114]. This method of incorporation ensures that only a dCTP can be inserted. Interestingly, the catalytic activity of Rev1 is not its most crucial function. In cells, Rev1 is required for tolerance of many DNA lesions, even though biochemically, Rev1 does not support TLS [115]. These findings suggest a role for Rev1 that regulates other polymerase activities. Indeed, Rev1 interacts with Pols η, κ, ι, and Rev7 as a scaffolding protein in response to exogenous damage [116,117]. Human Pol ζ consists of four subunits: the catalytic subunit (Rev3), an accessory subunit (Rev7) and two subunits shared with the replicative Pol δ [118]. The catalytic subunit of the Pol ζ holoenzyme can function alone, but its efficiency is enhanced when in complex with the other subunits [119,120]. Rev3 lacks a proofreading domain, making it error-prone [121]. A major known function of Pol ζ is its role in promoting mutagenesis. In mouse cells, decreased expression of *REV3* reduces UV-induced mutagenesis, but does not affect UV sensitivity [122]. Reducing *REV3* levels in lung tumor cells resulted in enhanced tumor cell killing by cisplatin and reduced therapy-induced mutagenesis [111].

### 3.1. Specialized Polymerases and Common Fragile Site Replication

Specialized Pols η, κ and ζ have been implicated in maintaining CFS stability. Pol η is present at the replication fork in unperturbed human cells [123]. Jean Sebastian-Hoffman and colleagues published a series of papers demonstrating that Pol η is required to maintain genomic stability at CFSs and prevent under-replicated DNA. Pol η-deficient human cells display increased formation of spontaneous chromosomal abnormalities and CFS breakage, suggesting that Pol η is important for CFS stability during unperturbed DNA replication [124]. Pol η-deficiency enhanced the formation of RPA foci and 53BP1 nuclear bodies, indicating the presence of under-replicated DNA [125]. Additionally, using chromatin immunoprecipitation and Pol η expression constructs, Pol η was found to be enriched at FRA7D and FRA16D CFS loci. Our laboratory used CFS-derived DNA template sequences to demonstrate biochemically that Pol η is more efficient than Pol δ for synthesis of CFS-derived DiToRS, including AT-rich repeats and quasipalindromes [125]. Recently, we used a dual-polymerase *in vitro* model and demonstrated directly that Pol η can take over DNA synthesis when the replicative Pol δ holoenzyme is stalled at CFS-derived DiToRS, particularly in the presence of aphidicolin [62]. Additionally, Pol η may participate in HR-associated mechanisms to restart replication forks stalled within CFS, due to its association with other proteins known to affect CFS stability, such as RAD51, BRCA2 and PALB2 [98,126]. Together, these studies demonstrate that Pol η is recruited to CFS during unperturbed and stressed conditions to synthesize DiToRS, facilitating complete genome duplication and DNA repair.

Pol κ also has roles in maintaining fragile site stability. In vitro, Pol κ efficiently extends DNA templates through [A/T], [AT/TA], and quasipalindrome DiToRS that inhibit replicative polymerases [60,61]. Like Pol η, Pol κ can freely exchange with the Pol δ holoenzyme to complete DiToRS synthesis, particularly in the presence of aphidicolin [62]. Pol κ has a characteristic high accuracy for slippage errors during microsatellite synthesis, greater than that of replicative Pol δ [82,127]. Recently, Nussensweig and colleagues identified regions in the genome termed “early-replicating fragile sites” that are AT-rich rich and display a Pol κ mutational signature of nontemplated insertion errors within [A/T] repeats [10,128]. However, in cancer cells, *POLK* depletion causes instability at the FRA7H CFS locus [129]. Further studies are needed to determine the roles of Pol κ for cellular DiToRS replication.

Evidence for the ability of Pol ζ to maintain DiToRS stability comes from two independent groups using *Saccharomyces cerevisiae* and human cells. In yeast, Northam et al. showed that Pol ζ is important for replication of undamaged DNA [130]. Their later work revealed that Pol ζ is specifically recruited to hairpin-forming DiToRS that cause stalling of replicative polymerases [131]. Using human cancer cells, Bhat et al. found that knockdown of *REV3* enhanced mitotic defects including anaphase bridges, lagging chromosomes and chromosomal breakage at CFS, indicating that Pol ζ is important for CFS maintenance [132].

### 3.2. Specialized Polymerase Synthesis of G4 Motifs

Specialized Pols η and κ, and Rev1, are involved in processing G4-quadruplexes. Rev1 acts as a major mediator of G4-quadruplex synthesis by regulating histone recycling and polymerase exchange [133]. Rev1 deficiency leads to changes in histone modifications flanking the G4-motifs, resulting in loss of parental chromatin marks [134]. Moreover, Rev1 destabilizes G4-quadruplexes by acting in concert with helicases such as FANCJ, BLM, or WRN [135,136]. Sale and colleagues proposed a handoff model wherein Rev1, with its favorable binding to poly-dG sequences, binds to G4-quadruplexes and initiates DNA synthesis, followed by exchange with Pol η or κ [133]. *In vitro*, Pol η and κ favor synthesis utilizing G4-quadruplex DNA templates over B-DNA templates [137]. Rev1, Pol κ, and Pol η perform complementary biochemical activities, efficiently replicating different nucleotide positions flanking and within G4-quadruplexes [137,138,139]. Indirect evidence supports roles for Pols η and κ in cellular replication of G4 motifs. Treatment of Pol η or κ- deficient cells with the G4 stabilizing agent, telomestatin, increases DSBs at G-rich loci [140], and stabilization of G4 DNA structures in Pol κ-deficient HeLa cells decreases viability [140]. Recently, an unbiased proteomic analysis of telomeric DNA uncovered a novel role for Pol η in maintaining telomere stability via a process known as alternative lengthening of telomeres (ALT) [141]. Telomerase-deficient cancer cells can utilize ALT to maintain telomeric DNA by forming ALT-associated PML bodies to facilitate homology-directed repair. Pol η, but not Pol κ, is co-localized to such bodies to resolve D-loops in cooperation with Pol δ. More studies are needed to understand why Pol η is specifically required for telomere synthesis.

The human mitochondrial (mt) genome also has sequences with non-B DNA forming potential, including G4-forming sequences, that are associated with mitochondrial diseases, cancer and aging [142,143,144,145]. For example, mtDNA deletion breakpoints are associated with non-B DNA forming sequences [143] and G quadruplex structures [144,145]. However, unlike DiToRS in the nuclear genome, relatively little is known regarding the extent to which such sequences in human mtDNA represent DiToRS. Brosh and colleagues showed that Twinkle, the replicative mitochondrial helicase, is inefficient at unwinding specific G4 sequences found in the mtDNA [145], supporting the concept that the formation of G4 structures perturbs mitochondrial genome replication, leading to DNA strand breaks and deletions.

## 4. Specialized DNA Polymerases and the Replication Stress Response

Genome DiToRS can lead to replisome stalling and the persistence of ssDNA during synthesis. The long stretches of ssDNA are bound by replication protein A (RPA) and in turn, the Ataxia telangiectasia and Rad3-related (ATR) protein kinase and its binding partner, ATR-interacting protein (ATRIP), are localized to the RPA-bound ssDNA. This causes chromatin localization of DNA topoisomerase 2-binding protein 1 (TOPBP1), an allosteric activator of ATR-ATRIP phosphorylation activity. Specifically, the interaction between TOPBP1 and DNA polymerase α-primase mediates recruitment of Rad9-Rad1-Hus1 (9-1-1) complex onto stalled forks [146]. Pol κ also plays a role in activating the 9-1-1 complex by interacting with Rad9, and is required for maintenance of genome stability and recovery from replication stress [147]. In response to mitomycin C-induced interstrand crosslinks, Rev1 functions to assemble the ATR/ATRIP and 9-1-1 complex [148].

ATR phosphorylation of Chk1 mediates the phosphorylation and repression of factors that slow down cell cycle progression (e.g., WEE1) and induce cell cycle arrest (e.g., CDC25A/C). Moreover, Chk1 orchestrates the inhibition of origin firing at new replication factories while simultaneously activating dormant origins within existing replication factories to prevent under-replicated DNA and genome instability [149]. ATR modulates the functions of numerous repair proteins involved in DNA unwinding (WRN, BLM) [150], homologous recombination [151], the Fanconi Anemia pathway [152,153], and the TLS pathway [154,155]. Consequently, ATR deficient (Seckel syndrome) cells display spontaneously increased CFS breakage in the absence of DNA damaging treatments [156]. ATR also is an important regulator and mediator of DNA polymerase activity and localization. Pol η is directly phosphorylated by ATR [154]. ATR-mediated phosphorylation of Pol η in response to UV damage, cisplatin and gemcitabine treatment leads to Pol η chromatin localization [157]. BPDE treatment of lung cancer cells results in ATR/Chk1-mediated recruitment of Pol κ via Rad18 and the subsequent monoubiquitination of PCNA, and inhibition of ATR/Chk1 signaling prevents the interaction between Pol κ and PCNA [155]. Additionally, chronic replication stress and endogenous DNA damage may deplete cellular RPA pools, leading to unprotected ssDNA and activation of the DNA damage response and cell death/senescence pathways controlled by the p53 and ATM/Chk2 [5,158]. Together, ATR, ATM, and p53 act in concert to maintain genome integrity and determine cell fate. Because of this, ATR, ATM, and p53 pathways are often under selective pressure to be altered or mutated during cancer cell evolution in order bypass tumor suppressive mechanisms.

While the cellular effects of replication stress induced by exogenous agents have been studied extensively, the discovery of physiologically-relevant models of replication stress was crucial to understanding mechanisms of genome instability during carcinogenesis. Di Micco et al. and Bartkova et al. first showed that oncogene activation induced replication stress which led to activation of the DNA damage response and senescence [159,160]. Moreover, hyper-replication was accompanied by increased origin firing and partly replicated DNA which were reminiscent of aphidicolin-treated cells [159]. Indeed, Miron et al. later showed that oncogene overexpression causes CFS instability similar to aphidicolin-treated cells [161]. However, while some overlapping regions of CFS instability were observed between oncogenes and aphidicolin, different oncogenes have a unique landscape of fragile sites, presumably caused by the different mechanisms of oncogenic stress. These studies paved the way for more than a decade of research on the sources of oncogenic replication stress.

## 5. Oncogenic Replication Stress Mechanisms

Oncogenes control a variety of physiological processes that are vital to cellular homeostasis including proliferation, apoptosis, epigenetics, metabolism, cell cycle regulation, transcription, DNA replication and DNA damage repair. For reviews on the functions of different oncogenes, refer to references [162,163]. Oncogene activation in pre-neoplastic cells causes genome instability preferentially at CFS loci [2,164,165,166]. The current paradigm of oncogene-mediated genome instability and tumorigenesis posits that excessive proliferative signaling leads to persistent replication stress, activation of the DNA damage response, and cellular senescence or programmed cell death, all of which are fail-safe mechanisms that shut down cellular proliferation. However, this presents a selective pressure for tumor cells to acquire mutations that allow bypass of cell cycle arrest and continue proliferation. In the presence of oncogene addiction, the replication stress response is constitutively active in cancer cells to alleviate the constant obstacles to genome replication, such as altered origin firing and nucleotide depletion [167,168,169].

Currently, 27 oncogenes have been studied for their impact on replication stress and each have distinct mechanisms [170]. Here, we focus on *Ras*, *CCNE1* (Cyclin E), and *c-Myc* which are, arguably, the three most studied oncogenes in the field of replication stress. We will highlight the collaboration between c-*Myc* and *Ras* or *CCNE1*, and how DiToRS replication may play a role in neoplastic transformation.

### 5.1. Balancing Cell Proliferation, Apoptosis, and Cell Death

Ras, Cyclin E and c-Myc proteins are all signaling hubs connected by upstream and downstream mitogenic pathways. Ras family members (H-Ras, K-Ras, N-Ras) affect signaling pathways downstream of the oncogenic MAPK pathway, including the Raf/MEK/ERK and PI3K/Akt pathways [171]. Constitutive activation of Ras signaling promotes expression of several growth factors and causes sustained growth and inhibition of apoptosis [172,173]. Cyclin E is a cell cycle regulator that dictates the G1/S transition and S phase progression. Hyperactivation of Cyclin E/CDK2 causes premature entry into S phase, which can be detrimental to genome integrity [174]. Moreover, Cyclin E can inhibit the pro-apoptotic FOXO1 transcriptional factor, thereby increasing proliferation [175]. The Myc family includes three oncoproteins (c-Myc, l-Myc, and n-Myc) [176]. These helix-loop-helix leucine zipper transcription factors are downstream of many mitogenic and signaling pathways that activate a plethora of cellular processes including metabolism, differentiation, cell size and pluripotency [177]. The impact of oncogene activation on cell fate (e.g., apoptosis versus cell proliferation) is dictated by the intra- and extracellular environment. For example, high levels of c-Myc promotes apoptosis under limiting growth factor conditions, whereas cells with plentiful growth factors respond with rapid proliferation [178]. Thus, it is likely that normal cells generate a pro-apoptotic program in response to c-Myc activation whereas transformed cells only respond to its proliferative signals.

The tumor protective mechanisms apoptosis and senescence are robust responses that prevent a single oncogene activation from promoting tumorigenesis. Senescence is a state of cell cycle arrest in which cells shut down proliferation but retain metabolic activity [179]. For years, senescence was referred to as an irreversible state of arrest. However, recent studies suggest that, in some cases, alterations in CDKs, p53, p16(INK4A), or Rb can reverse senescence and restore cell cycle progression [180,181]. These findings suggest that different oncogenes may collaborate to reverse or bypass the onset of senescence. Indeed, co-expression of c-Myc suppresses Ras-induced senescence via Cdk2 activity [182].

### 5.2. Regulation of DNA Replication and S Phase

**Figure 2:**
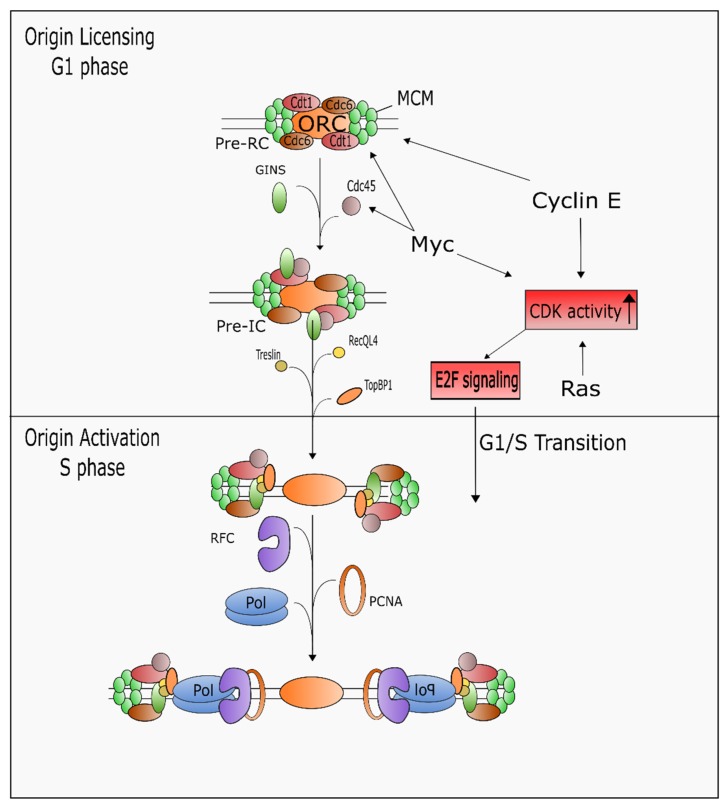
Oncogenes induce origin licensing and E2F transcriptional activity to drive G1/S transition and DNA hyper-replication. Myc and Cyclin E directly increase origin licensing by facilitating recruitment of pre-initiation complex (pre-IC) factors, including Cdt1, Cdc6, and MCMs. High levels of Ras, Myc or Cyclin E lead to increased CDK phosphorylation activity and E2F signaling that promotes the G1/S transition and initiation of DNA synthesis.

DNA replication is a highly coordinated process that precisely duplicates DNA once during each cell cycle [183]. Initiation of DNA synthesis includes two stages: origin licensing and activation (Figure 2). Licensing begins with ORC binding in late mitosis to G1 and requires little to no CDK activity, whereas activation occurs only after entry into S-phase and requires high CDK activity. Origin licensing proceeds through a series of steps, beginning with recruitment of pre-replicative complex (pre-RC) proteins (Cdt1 and Cdc6) and the minichromosome maintenance 2–7 (MCM2–7) helicase. Subsequently, the pre-initiation (pre-IC) complex is formed by Cdk- and Dbf4-dependent kinase (DDK)-mediated phosphorylation and activation of the MCM helicases. This mediates recruitment of MCM10 which is important for the recruitment of Cdc45 and GINS complex to form the CMG complex (Cdc45/MCM2-7/GINS) [184,185,186]. Origin activation occurs after phosphorylation of the CMG complex splits the MCM proteins into two separate hexamers for the two bi-directional replisomes. This is accompanied by the recruitment of several replication proteins, including TopBP1. Simultaneously, the clamp loader RFC and sliding clamp PCNA are recruited, followed by CMG- and MCM10-mediated recruitment and interaction of the replicative polymerases δ, ε, and α for the initiation of synthesis [187,188].

The highly complex process of origin activation is readily altered by oncogene activation (Figure 2). One of the main sources of replication stress is the dysregulation of origin usage via altered origin activation or inappropriate re-firing of origins. Indeed, overexpression of Cdt1 or Cdc6 induces replication stress [189,190]. Moreover, depletion of pre-RC proteins in the presence of oncogenes cyclin E, H-Ras, K-Ras, or c-Myc sensitizes cancer cells to replication stress-inducing agents [191,192]. The Ras, c-Myc and cyclin E oncogenes also alter CDK activity which, in turn, causes excessive origin licensing and activation. Depending on the cellular context, Cyclin E can either inhibit pre-RC formation via impairment of MCM loading, or promote pre-RC formation, forcing the G1/S phase transition [193,194]. Myc overexpression creates DNA replication stress in two ways: by regulating origin activation and by directly promoting the G1/S transition. Myc facilitates pre-RC formation by interacting with Orc1/2, Cdc45, TOPBP1, and MCMs, and by transcriptionally regulating Cdt1 expression [195,196]. Myc also promotes tumorigenesis by directly activating the replication stress response. Hypomorphic levels of ATR prevents the development of Myc-induced lymphomas and pancreatic tumors [197], and Myc activates transcription of *CHEK*1, *CHEK*2, and *WRN* genes [198,199]. Re-firing of origins, also known as re-replication, is caused by aberrant expression of Cdt1 and Cdc6 as well as by Cyclin E and c-Myc overexpression [180,200]. Re-replication leads to genome instability, in part, through increased head-to-tail collisions between newly formed and existing replication forks [201].

Aberrant regulation of the G1/S transition is a common feature of oncogenes that creates replication stress, and the activation/inactivation of oncogenes and tumor suppressor genes that influence the G1/S transition are found in most human cancers [202]. Recently, Macheret and Halazonetis found that overexpression of cyclin E and and c-Myc leads to an increase of fired DNA replication origins within highly transcribed intragenic regions [203]. Moreover, they found increased replication stress and collapsed forks resulting in double stranded breaks at these newly fired origins, which was alleviated by inhibiting transcription. These data suggest that during oncogene activation, the shortened G1 phase may leave transcription of G1 and S phase genes unfinished, which leads to increased collisions with the replisome.

### 5.3. Alterations in Metabolism

Ras and c-Myc have extensive connections to metabolic pathways. Oncogenic Ras promotes metabolic reprogramming of the cell through induction of anabolic glycolysis and autophagy-mediated protein recycling to support its increase in cell proliferation and biomass [204,205,206]. As a transcription factor, c-Myc can directly increase transcription of genes involved in nucleotide biosynthesis [207], mitochondrial biogenesis [208], glycolysis [209], and glutaminolysis [210]. Because of the overarching reach of c-Myc on metabolism, c-Myc often collaborates with other oncogenes to drive tumorigenesis. In fact, c-Myc can be spontaneously activated by oncogenic Ras to promote transformation and increase cellular metabolism [211,212,213]. Recently, Myc and Ras overexpression were shown to have distinct metabolic consequences [214], although both Myc and Ras overexpression led to increased replicative stress. Ras enhanced metabolic activity of glycolysis and oxygen consumption that correlated with slower DNA replication fork progression, whereas Myc-induced less drastic metabolic changes but increased oxidative stress.

Oncogene-induced changes in metabolism also may lead to replication stress via depletion of dNTP nucleotide precursors. Alteration of dNTP pools is a well-known physiologic source of replication stress in cancer cells [8,215], and dNTP pools are crucial for DNA polymerase biochemistry and fidelity [216,217,218]. dNTPs are synthesized via ribonucleotide reductase (RNR), which is composed of a catalytic (RRM1) and two regulatory (RRM2, RRM2B) domains. *RRM2* expression is specifically increased during S-phase to regulate dNTP levels, and oncogene activation represses *RRM2* gene expression [219,220]. Knockdown of ATM rescued Ras-induced dNTP depletion and senescence [213] and was accompanied by upregulation of c-Myc-mediated nucleotide biosynthesis. Disruptions of *de novo* dNTP pool homeostasis also can be caused by metabolic changes, such as low glutamine levels [221]. Expression of the HPV E6/E7 oncoproteins and cyclin E cause replication stress, altered dNTP levels, and genome instability at DiToRS [222]. Interestingly, co-expression of c-Myc in cells with cyclin E or E6/E7 led to upregulation of nucleotide biosynthesis-associated genes [222,223]. Together, these results further highlight the role of c-Myc in cooperating with other oncogenes and the importance of maintaining dNTP homeostasis and metabolic reprogramming during oncogene-induced tumorigenesis.

## 6. Tolerance of Oncogenic Replication Stress via Specialized DNA Polymerases

The impact of oncogenic replication stress on cellular transformation is well established; however, the precise mechanisms by which cells tolerate oncogenic-replication stress are less clear. DNA polymerase functions are central to preventing replication stress, especially during DiToRS synthesis. Two recent studies have elucidated a role for specialized DNA polymerases η and κ in the cellular tolerance of oncogenic replication stress. Yang et al. showed that overexpression of cyclin E and H-Ras in human cells caused Pol κ re-localization to chromatin in a Rad18-dependent manner [215]. Overexpression of cyclin E or Ras was accompanied by increased PCNA monoubiquitination, which could be suppressed by treatment with roscovitine, a CDK2 inhibitor. Using the WEE1 inhibitor, MK-1775, the authors showed that Pol κ is required to prevent ssDNA accumulation and decreased cell viability induced by aberrant CDK2 activity. Moreover, cyclin E, but not Cdt1 or Cdc6, overexpression increased chromatin binding of Cdc45 and PCNA monoubiquitination, suggesting that Rad18 activity is mediated by origin activation but not by origin licensing. Importantly, activation of Rad18-mediated Pol κ activity is attenuated by p53 in cells with aberrant Cdk2 activity. Because p53 is often mutated in tumors, these results suggest that specialized polymerase activity may be important for bypass of oncogene-induced senescence or cell death. Kurashima et al. uncovered a role for Pol η in alleviating Myc-induced replication stress [224]. They proposed that Pol η directly suppresses Myc-induced replication stress by mediating fork progression. Depletion of Cdc45 decreased Myc-induced replication stress, suggesting origin hyper-activation was the source of oncogenic replication stress in this model. Myc activation in Pol η-deficient and mutant cells synergistically increased double strand break formation compared to cells with proficient Pol η. These two papers suggest that the role of Pols η and κ in the tolerance of oncogenic stress is context dependent. It is intriguing that H-Ras and cyclin E are more reliant on Pol κ, whereas Myc relies on Pol η. Moreover, the roles of Rev1 and/or Pol ζ in alleviating oncogenic replication stress are still unknown.

## 7. Perspective

**Figure 3:**
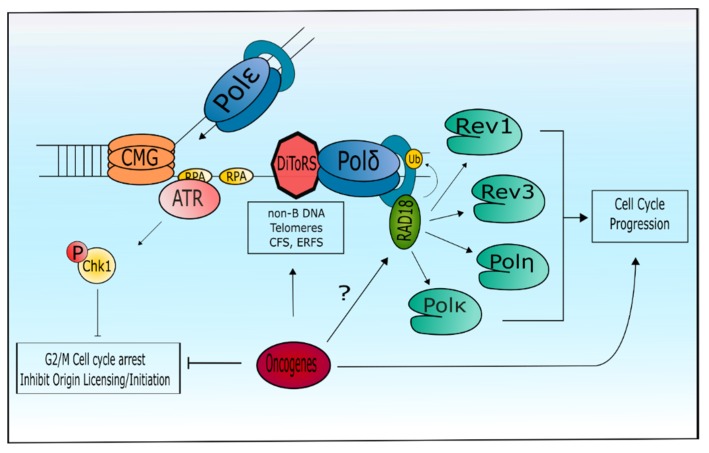
Conceptual framework for the role of specialized polymerases in oncogene induced replication stress. Oncogene activation promotes DNA replication thereby increasing replication fork encounters with DiToRS. Fork stalling activates the ATR/Chk1 axis and the subsequent recruitment of specialized DNA polymerase to resolve impediments. Rad18-mediated ubiquitination of PCNA is responsible, in part, for the recruitment of specialized DNA polymerase for distinct forms of DiToRS depending on the oncogenic cellular context. Engagement of specialized polymerases allows fork progression past DiToRS and continued cell cycle progression.

Although our understanding of the fate of the replication fork during oncogenic replication stress is becoming clearer, much remains to be discovered by characterizing the effects of different oncogenes, unique DiToRS in the genome, and the precise mechanisms by which cells tolerate oncogene replication stress. We propose a conceptual framework wherein oncogene activation cooperates with replication forks stalled at DiToRS, leading to the recruitment of specialized DNA polymerases to resolve fork stalling and resume DNA elongation and cell cycle progression (Figure 3). One possible model is that different polymerases are required to tolerate distinct forms of replication stress or DiToRS obstacles. Rad18-mediated PCNA monubiquitination is one mechanism to promote recruitment of different specialized DNA polymerases, depending on oncogenic cellular context and the distinct forms of DiToRS. However, PCNA monubiquitination is not required for specialized/replicative polymerase exchange at DiToRS [62,225], so other regulators of DNA polymerases in the context of oncogene induced replication stress remain to be discovered. Additional studies are needed to determine whether other post-translational modifications of specialized polymerases, such as sumoylation or phosphorylation, are involved in engaging these polymerases during oncogene replication stress. Another open question is the extent to which specialized DNA polymerases can compensate for each other. Interestingly, Pol η deficient cells were able to resist the negative effects of CDK2 activation, possibly because Pol κ can localize to replication stressed loci without competing with Pol η [215]. Clearly, more studies are necessary to understand the precise mechanisms by which specialized polymerases promote tolerance of oncogenic replication stress. Finally, specialized polymerases may play a role in oncogene activation. *Myc* is of particular importance regarding DiToRS, due to the structure of its promoter region. The c-*Myc* promoter consists of sequences that can form H-DNA, Z-DNA, and G4 quadruplexes [226,227,228,229]. It is therefore tempting to speculate that the deregulation of specialized polymerases may cause spontaneous amplification of *c-Myc* or other cancer-associated genes encoding DiToRS. Interestingly, human tumors show a bias for *POLH* amplification but *POLK* deletion (Figure 1A), raising the possibility that alterations in these two DNA polymerases may favor bypass of senescence and promote cellular transformation. Identifying which DNA polymerases are crucial for cells to tolerate the activation of different oncogenes will provide insight into carcinogenesis and responses to therapy. With the advent of exploiting DNA repair and replication stress pathways as cancer therapies, it is crucial to understand how specialized polymerases can contribute to chemoresistance and tumor relapse. Recently, we have found that exogenous replicative stress inducers increase *POLH* mRNA and Pol η protein expression to prevent cell cycle arrest [230]. Moreover, inhibiting ATR in Pol η-depleted or deficient cells undergoing replicative stress resulted in synthetic lethality, suggesting a possible therapeutic option. Importantly, ATR and Chk1 inhibitors are a promising avenue of drug therapy with both currently undergoing clinical trials [231]. Thus, targeting specific tumors based on expression of specialized DNA polymerases to generate synthetic lethality undergoing “oncogene addiction” may be a viable therapeutic option in the future.

## Abbreviations

CFS	Common Fragile Site
DiToRS	Difficult-to-Replicate Sequences
Non-B DNA	DNA secondary structures other than B form

## References

[1] Macheret M., Halazonetis T.D. (2015). DNA replication stress as a hallmark of cancer. Annu. Rev. Pathol..

[2] Tsantoulis P.K., Kotsinas A., Sfikakis P.P., Evangelou K., Sideridou M., Levy B., Mo L., Kittas C., Wu X.R., Papavassiliou A.G. (2008). Oncogene-induced replication stress preferentially targets common fragile sites in preneoplastic lesions. A genome-wide study. Oncogene.

[3] Halazonetis T.D., Gorgoulis V.G., Bartek J. (2008). An oncogene-induced DNA damage model for cancer development. Science.

[4] Negrini S., Gorgoulis V.G., Halazonetis T.D. (2010). Genomic instability—An evolving hallmark of cancer. Nat. Rev. Mol. Cell Biol..

[5] Zeman M.K., Cimprich K.A. (2014). Causes and consequences of replication stress. Nat. Cell Biol..

[6] Bochman M.L., Paeschke K., Zakian V.A. (2012). DNA secondary structures: Stability and function of G-quadruplex structures. Nat. Rev. Genet..

[7] Mirkin E.V., Mirkin S.M. (2007). Replication fork stalling at natural impediments. Microb. Mol. Biol. Rev..

[8] Técher H., Koundrioukoff S., Nicolas A., Debatisse M. (2017). The impact of replication stress on replication dynamics and DNA damage in vertebrate cells. Nat. Rev. Genet..

[9] Le Tallec B., Koundrioukoff S., Wilhelm T., Letessier A., Brison O., Debatisse M. (2014). Updating the mechanisms of common fragile site instability: How to reconcile the different views?. Cell. Mol. Life Sci..

[10] Barlow J.H., Faryabi R.B., Callén E., Wong N., Malhowski A., Chen H.T., Gutierrez-Cruz G., Sun H.W., McKinnon P., Wright G. (2013). Identification of early replicating fragile sites that contribute to genome instability. Cell.

[11] Mirkin S.M. (2007). Expandable DNA repeats and human disease. Nature.

[12] Sfeir A., Kosiyatrakul S.T., Hockemeyer D., MacRae S.L., Karlseder J., Schildkraut C.L., de Lange T. (2009). Mammalian telomeres resemble fragile sites and require TRF1 for efficient replication. Cell.

[13] Zhang H., Freudenreich C.H. (2007). An AT-rich sequence in human common fragile site FRA16D causes fork stalling and chromosome breakage in S. cerevisiae. Mol. Cell.

[14] Hamperl S., Cimprich K.A. (2016). Conflict Resolution in the Genome: How Transcription and Replication Make It Work. Cell.

[15] Vaisman A., Woodgate R. (2017). Translesion DNA polymerases in eukaryotes: What makes them tick?. Crit. Rev. Biochem. Mol. Biol..

[16] Burrell R.A., McClelland S.E., Endesfelder D., Groth P., Weller M.C., Shaikh N., Domingo E., Kanu N., Dewhurst S.M., Gronroos E. (2013). Replication stress links structural and numerical cancer chromosomal instability. Nature.

[17] de Koning A.P., Gu W., Castoe T.A., Batzer M.A., Pollock D.D. (2011). Repetitive elements may comprise over two-thirds of the human genome. PLoS Genet..

[18] Watson J.D., Crick F.H. (2003). A structure for deoxyribose nucleic acid. 1953. Nature.

[19] Wang A.H., Quigley G.J., Kolpak F.J., Crawford J.L., van Boom J.H., van der Marel G., Rich A. (1979). Molecular structure of a left-handed double helical DNA fragment at atomic resolution. Nature.

[20] Mirkin S.M., Lyamichev V.I., Drushlyak K.N., Dobrynin V.N., Filippov S.A., Frank-Kamenetskii M.D. (1987). DNA H form requires a homopurine-homopyrimidine mirror repeat. Nature.

[21] Schroth G.P., Ho P.S. (1995). Occurrence of potential cruciform and H-DNA forming sequences in genomic DNA. Nucleic Acids Res..

[22] Sen D., Gilbert W. (1988). Formation of parallel four-stranded complexes by guanine-rich motifs in DNA and its implications for meiosis. Nature.

[23] Parkinson G.N., Lee M.P., Neidle S. (2002). Crystal structure of parallel quadruplexes from human telomeric DNA. Nature.

[24] Koo H.S., Wu H.M., Crothers D.M. (1986). DNA bending at adenine. thymine tracts. Nature.

[25] Huppert J.L. (2010). Structure, location and interactions of G-quadruplexes. FEBS J..

[26] Inagaki H., Ohye T., Kogo H., Kato T., Bolor H., Taniguchi M., Shaikh T.H., Emanuel B.S., Kurahashi H. (2009). Chromosomal instability mediated by non-B DNA: Cruciform conformation and not DNA sequence is responsible for recurrent translocation in humans. Genome Res..

[27] Bissler J.J. (1998). DNA inverted repeats and human disease. Front. Biosci..

[28] Thys R.G., Lehman C.E., Pierce L.C., Wang Y.H. (2015). DNA secondary structure at chromosomal fragile sites in human disease. Curr. Genom..

[29] Wang H., Pierce L.J., Spangrude G.J. (2006). Distinct roles of IL-7 and stem cell factor in the OP9-DL1 T-cell differentiation culture system. Exp. Hematol..

[30] Wang G., Carbajal S., Vijg J., DiGiovanni J., Vasquez K.M. (2008). DNA structure-induced genomic instability in vivo. J. Natl. Cancer Inst..

[31] McGinty R.J., Mirkin S.M. (2018). Cis- and Trans-Modifiers of Repeat Expansions: Blending Model Systems with Human Genetics. Trends Genet..

[32] Lee J.A., Carvalho C.M., Lupski J.R. (2007). A DNA replication mechanism for generating nonrecurrent rearrangements associated with genomic disorders. Cell.

[33] Colnaghi R., Carpenter G., Volker M., O’Driscoll M. (2011). The consequences of structural genomic alterations in humans: Genomic disorders, genomic instability and cancer. Semin. Cell Dev. Biol..

[34] Bacolla A., Tainer J.A., Vasquez K.M., Cooper D.N. (2016). Translocation and deletion breakpoints in cancer genomes are associated with potential non-B DNA-forming sequences. Nucleic Acids Res..

[35] Burrow A.A., Williams L.E., Pierce L.C., Wang Y.H. (2009). Over half of breakpoints in gene pairs involved in cancer-specific recurrent translocations are mapped to human chromosomal fragile sites. BMC Genom..

[36] Zhao J., Wang G., Del Mundo I.M., McKinney J.A., Lu X., Bacolla A., Boulware S.B., Zhang C., Zhang H., Ren P. (2018). Distinct Mechanisms of Nuclease-Directed DNA-Structure-Induced Genetic Instability in Cancer Genomes. Cell Rep..

[37] Zhao J., Bacolla A., Wang G., Vasquez K.M. (2010). Non-B DNA structure-induced genetic instability and evolution. Cell. Mol. Life Sci..

[38] Biscotti M.A., Olmo E., Heslop-Harrison J.S. (2015). Repetitive DNA in eukaryotic genomes. Chromosome Res..

[39] Gemayel R., Vinces M.D., Legendre M., Verstrepen K.J. (2010). Variable tandem repeats accelerate evolution of coding and regulatory sequences. Annu. Rev. Genet..

[40] Li Y.C., Korol A.B., Fahima T., Beiles A., Nevo E. (2002). Microsatellites: Genomic distribution, putative functions and mutational mechanisms: A review. Mol. Ecol..

[41] Durkin S.G., Glover T.W. (2007). Chromosome fragile sites. Annu. Rev. Genet..

[42] Zlotorynski E., Rahat A., Skaug J., Ben-Porat N., Ozeri E., Hershberg R., Levi A., Scherer S.W., Margalit H., Kerem B. (2003). Molecular basis for expression of common and rare fragile sites. Mol. Cell. Biol..

[43] Helmrich A., Stout-Weider K., Hermann K., Schrock E., Heiden T. (2006). Common fragile sites are conserved features of human and mouse chromosomes and relate to large active genes. Genome Res..

[44] Schoder C., Liehr T., Velleuer E., Wilhelm K., Blaurock N., Weise A., Mrasek K. (2010). New aspects on chromosomal instability: Chromosomal break-points in Fanconi anemia patients co-localize on the molecular level with fragile sites. Int. J. Oncol..

[45] Bignell G.R., Greenman C.D., Davies H., Butler A.P., Edkins S., Andrews J.M., Buck G., Chen L., Beare D., Latimer C. (2010). Signatures of mutation and selection in the cancer genome. Nature.

[46] Hussein S.M., Batada N.N., Vuoristo S., Ching R.W., Autio R., Narva E., Ng S., Sourour M., Hamalainen R., Olsson C. (2011). Copy number variation and selection during reprogramming to pluripotency. Nature.

[47] Dall K.L., Scarpini C.G., Roberts I., Winder D.M., Stanley M.A., Muralidhar B., Herdman M.T., Pett M.R., Coleman N. (2008). Characterization of naturally occurring HPV16 integration sites isolated from cervical keratinocytes under noncompetitive conditions. Cancer Res..

[48] Bester A.C., Schwartz M., Schmidt M., Garrigue A., Hacein-Bey-Abina S., Cavazzana-Calvo M., Ben-Porat N., Von Kalle C., Fischer A., Kerem B. (2006). Fragile sites are preferential targets for integrations of MLV vectors in gene therapy. Gene Ther..

[49] Dillon L.W., Burrow A.A., Wang Y.H. (2010). DNA instability at chromosomal fragile sites in cancer. Curr. Genom..

[50] Lukusa T., Fryns J.P. (2008). Human chromosome fragility. Biochim. Biophys. Acta.

[51] Letessier A., Millot G.A., Koundrioukoff S., Lachagès A.M., Vogt N., Hansen R.S., Malfoy B., Brison O., Debatisse M. (2011). Cell-type-specific replication initiation programs set fragility of the FRA3B fragile site. Nature.

[52] Ozeri-Galai E., Lebofsky R., Rahat A., Bester A.C., Bensimon A., Kerem B. (2011). Failure of origin activation in response to fork stalling leads to chromosomal instability at fragile sites. Mol. Cell.

[53] Palakodeti A., Lucas I., Jiang Y., Young D.J., Fernald A.A., Karrison T., Le Beau M.M. (2010). Impaired replication dynamics at the FRA3B common fragile site. Hum. Mol. Genet..

[54] Helmrich A., Ballarino M., Tora L. (2011). Collisions between replication and transcription complexes cause common fragile site instability at the longest human genes. Mol. Cell.

[55] Fungtammasan A., Walsh E., Chiaromonte F., Eckert K.A., Makova K.D. (2012). A genome-wide analysis of common fragile sites: What features determine chromosomal instability in the human genome?. Genome Res..

[56] Mishmar D., Rahat A., Scherer S.W., Nyakatura G., Hinzmann B., Kohwi Y., Mandel-Gutfroind Y., Lee J.R., Drescher B., Sas D.E. (1998). Molecular characterization of a common fragile site (FRA7H) on human chromosome 7 by the cloning of a simian virus 40 integration site. Proc. Natl. Acad. Sci. USA.

[57] Madireddy A., Kosiyatrakul S.T., Boisvert R.A., Herrera-Moyano E., Garcia-Rubio M.L., Gerhardt J., Vuono E.A., Owen N., Yan Z., Olson S. (2016). FANCD2 Facilitates Replication through Common Fragile Sites. Mol. Cell.

[58] Burrow A.A., Marullo A., Holder L.R., Wang Y.H. (2010). Secondary structure formation and DNA instability at fragile site FRA16B. Nucleic Acids Res..

[59] Shah S.N., Opresko P.L., Meng X., Lee M.Y., Eckert K.A. (2010). DNA structure and the Werner protein modulate human DNA polymerase Delta-dependent replication dynamics within the common fragile site FRA16D. Nucleic Acids Res..

[60] Walsh E., Wang X., Lee M.Y., Eckert K.A. (2013). Mechanism of replicative DNA polymerase Delta pausing and a potential role for DNA polymerase Kappa in common fragile site replication. J. Mol. Biol..

[61] Hile S.E., Eckert K.A. (2008). DNA polymerase kappa produces interrupted mutations and displays polar pausing within mononucleotide microsatellite sequences. Nucleic Acids Res..

[62] Barnes R.P., Hile S.E., Lee M.Y., Eckert K.A. (2017). DNA polymerases Eta and Kappa exchange with the polymerase delta holoenzyme to complete common fragile site synthesis. DNA Repair (Amst).

[63] Usdin K., House N.C., Freudenreich C.H. (2015). Repeat instability during DNA repair: Insights from model systems. Crit. Rev. Biochem. Mol. Biol..

[64] Usdin K., Woodford K.J. (1995). CGG repeats associated with DNA instability and chromosome fragility form structures that block DNA synthesis in vitro. Nucleic Acids Res..

[65] Weitzmann M.N., Woodford K.J., Usdin K. (1996). The development and use of a DNA polymerase arrest assay for the evaluation of parameters affecting intrastrand tetraplex formation. J. Biol. Chem..

[66] Pelletier R., Krasilnikova M.M., Samadashwily G.M., Lahue R., Mirkin S.M. (2003). Replication and expansion of trinucleotide repeats in yeast. Mol. Cell. Biol..

[67] Voineagu I., Surka C.F., Shishkin A.A., Krasilnikova M.M., Mirkin S.M. (2009). Replisome stalling and stabilization at CGG repeats, which are responsible for chromosomal fragility. Nat. Struct. Mol. Biol..

[68] Burge S., Parkinson G.N., Hazel P., Todd A.K., Neidle S. (2006). Quadruplex DNA: Sequence, topology and structure. Nucleic Acids Res..

[69] Dilley R.L., Verma P., Cho N.W., Winters H.D., Wondisford A.R., Greenberg R.A. (2016). Break-induced telomere synthesis underlies alternative telomere maintenance. Nature.

[70] Lormand J.D., Buncher N., Murphy C.T., Kaur P., Lee M.Y., Burgers P., Wang H., Kunkel T.A., Opresko P.L. (2013). DNA polymerase delta stalls on telomeric lagging strand templates independently from G-quadruplex formation. Nucleic Acids Res..

[71] De S., Michor F. (2011). DNA secondary structures and epigenetic determinants of cancer genome evolution. Nat. Struct. Mol. Biol..

[72] Wang G., Vasquez K.M. (2004). Naturally occurring H-DNA-forming sequences are mutagenic in mammalian cells. Proc. Natl. Acad. Sci. USA.

[73] Hile S.E., Eckert K.A. (2004). Positive correlation between DNA polymerase alpha-primase pausing and mutagenesis within polypyrimidine/polypurine microsatellite sequences. J. Mol. Biol..

[74] Krasilnikova M.M., Mirkin S.M. (2004). Replication stalling at Friedreich’s ataxia (GAA)n repeats in vivo. Mol. Cell. Biol..

[75] Chandok G.S., Patel M.P., Mirkin S.M., Krasilnikova M.M. (2012). Effects of Friedreich’s ataxia GAA repeats on DNA replication in mammalian cells. Nucleic Acids Res..

[76] Follonier C., Oehler J., Herrador R., Lopes M. (2013). Friedreich’s ataxia-associated GAA repeats induce replication-fork reversal and unusual molecular junctions. Nat. Struct. Mol. Biol..

[77] Casper A.M., Greenwell P.W., Tang W., Petes T.D. (2009). Chromosome aberrations resulting from double-strand DNA breaks at a naturally occurring yeast fragile site composed of inverted ty elements are independent of Mre11p and Sae2p. Genetics.

[78] Seier T., Padgett D.R., Zilberberg G., Sutera V.A., Toha N., Lovett S.T. (2011). Insights into mutagenesis using Escherichia coli chromosomal lacZ strains that enable detection of a wide spectrum of mutational events. Genetics.

[79] Voineagu I., Narayanan V., Lobachev K.S., Mirkin S.M. (2008). Replication stalling at unstable inverted repeats: Interplay between DNA hairpins and fork stabilizing proteins. Proc. Natl. Acad. Sci. USA.

[80] Lu S., Wang G., Bacolla A., Zhao J., Spitser S., Vasquez K.M. (2015). Short Inverted Repeats Are Hotspots for Genetic Instability: Relevance to Cancer Genomes. Cell Rep..

[81] Waters L.S., Minesinger B.K., Wiltrout M.E., D’Souza S., Woodruff R.V., Walker G.C. (2009). Eukaryotic translesion polymerases and their roles and regulation in DNA damage tolerance. Microb. Mol. Biol. Rev..

[82] Hile S.E., Wang X., Lee M.Y., Eckert K.A. (2012). Beyond translesion synthesis: Polymerase κ fidelity as a potential determinant of microsatellite stability. Nucleic Acids Res..

[83] Sale J.E. (2013). Translesion DNA synthesis and mutagenesis in eukaryotes. Cold Spring Harb. Perspect. Biol..

[84] Barnes R., Eckert K. (2017). Maintenance of Genome Integrity: How Mammalian Cells Orchestrate Genome Duplication by Coordinating Replicative and Specialized DNA Polymerases. Genes (Basel).

[85] Bournique E., Dall’Osto M., Hoffmann J.S., Bergoglio V. (2018). Role of specialized DNA polymerases in the limitation of replicative stress and DNA damage transmission. Mutat. Res..

[86] Boyer A.S., Grgurevic S., Cazaux C., Hoffmann J.S. (2013). The human specialized DNA polymerases and non-B DNA: Vital relationships to preserve genome integrity. J. Mol. Biol..

[87] Cerami E., Gao J., Dogrusoz U., Gross B.E., Sumer S.O., Aksoy B.A., Jacobsen A., Byrne C.J., Heuer M.L., Larsson E. (2012). The cBio cancer genomics portal: An open platform for exploring multidimensional cancer genomics data. Cancer Discov..

[88] Gao J., Aksoy B.A., Dogrusoz U., Dresdner G., Gross B., Sumer S.O., Sun Y., Jacobsen A., Sinha R., Larsson E. (2013). Integrative analysis of complex cancer genomics and clinical profiles using the cBioPortal. Sci Signal.

[89] Ceppi P., Novello S., Cambieri A., Longo M., Monica V., Lo Iacono M., Giaj-Levra M., Saviozzi S., Volante M., Papotti M. (2009). Polymerase eta mRNA expression predicts survival of non-small cell lung cancer patients treated with platinum-based chemotherapy. Clin. Cancer Res..

[90] Zhou W., Chen Y.W., Liu X., Chu P., Loria S., Wang Y., Yen Y., Chou K.M. (2013). Expression of DNA translesion synthesis polymerase eta in head and neck squamous cell cancer predicts resistance to gemcitabine and cisplatin-based chemotherapy. PLoS ONE.

[91] Masutani C., Kusumoto R., Yamada A., Dohmae N., Yokoi M., Yuasa M., Araki M., Iwai S., Takio K., Hanaoka F. (1999). The XPV (xeroderma pigmentosum variant) gene encodes human DNA polymerase eta. Nature.

[92] Broughton B.C., Cordonnier A., Kleijer W.J., Jaspers N.G., Fawcett H., Raams A., Garritsen V.H., Stary A., Avril M.F., Boudsocq F. (2002). Molecular analysis of mutations in DNA polymerase eta in xeroderma pigmentosum-variant patients. Proc. Natl. Acad. Sci. USA.

[93] Masutani C., Kusumoto R., Iwai S., Hanaoka F. (2000). Mechanisms of accurate translesion synthesis by human DNA polymerase eta. EMBO J..

[94] Biertümpfel C., Zhao Y., Kondo Y., Ramón-Maiques S., Gregory M., Lee J.Y., Masutani C., Lehmann A.R., Hanaoka F., Yang W. (2010). Structure and mechanism of human DNA polymerase eta. Nature.

[95] Stary A., Kannouche P., Lehmann A.R., Sarasin A. (2003). Role of DNA polymerase eta in the UV mutation spectrum in human cells. J. Biol. Chem..

[96] Wang Y.C., Maher V.M., McCormick J.J. (1991). Xeroderma pigmentosum variant cells are less likely than normal cells to incorporate dAMP opposite photoproducts during replication of UV-irradiated plasmids. Proc. Natl. Acad. Sci. USA.

[97] Peña-Diaz J., Bregenhorn S., Ghodgaonkar M., Follonier C., Artola-Borán M., Castor D., Lopes M., Sartori A.A., Jiricny J. (2012). Noncanonical mismatch repair as a source of genomic instability in human cells. Mol. Cell.

[98] McIlwraith M.J., Mcllwraith M.J., Vaisman A., Liu Y., Fanning E., Woodgate R., West S.C. (2005). Human DNA polymerase eta promotes DNA synthesis from strand invasion intermediates of homologous recombination. Mol. Cell.

[99] Zeng X., Winter D.B., Kasmer C., Kraemer K.H., Lehmann A.R., Gearhart P.J. (2001). DNA polymerase eta is an A-T mutator in somatic hypermutation of immunoglobulin variable genes. Nat. Immunol..

[100] Rogozin I.B., Goncearenco A., Lada A.G., De S., Yurchenko V., Nudelman G., Panchenko A.R., Cooper D.N., Pavlov Y.I. (2018). DNA polymerase η mutational signatures are found in a variety of different types of cancer. Cell Cycle.

[101] Albertella M.R., Lau A., O’Connor M.J. (2005). The overexpression of specialized DNA polymerases in cancer. DNA Repair (Amst).

[102] Pan Q., Fang Y., Xu Y., Zhang K., Hu X. (2005). Down-regulation of DNA polymerases κ, η, ι, and ζ in human lung, stomach, and colorectal cancers. Cancer Lett..

[103] Pillaire M.J., Selves J., Gordien K., Gourraud P.A., Gouraud P.A., Gentil C., Danjoux M., Do C., Negre V., Bieth A. (2010). A ‘DNA replication’ signature of progression and negative outcome in colorectal cancer. Oncogene.

[104] Lone S., Townson S.A., Uljon S.N., Johnson R.E., Brahma A., Nair D.T., Prakash S., Prakash L., Aggarwal A.K. (2007). Human DNA polymerase Kappa encircles DNA: Implications for mismatch extension and lesion bypass. Mol. Cell.

[105] Ogi T., Lehmann A.R. (2006). The Y-family DNA polymerase κ (pol κ) functions in mammalian nucleotide-excision repair. Nat. Cell Biol..

[106] Zhang X., Lv L., Chen Q., Yuan F., Zhang T., Yang Y., Zhang H., Wang Y., Jia Y., Qian L. (2013). Mouse DNA polymerase Kappa has a functional role in the repair of DNA strand breaks. DNA Repair (Amst).

[107] Avkin S., Goldsmith M., Velasco-Miguel S., Geacintov N., Friedberg E.C., Livneh Z. (2004). Quantitative analysis of translesion DNA synthesis across a benzo[a]pyrene-guanine adduct in mammalian cells: The role of DNA polymerase kappa. J. Biol. Chem..

[108] Bi X., Slater D.M., Ohmori H., Vaziri C. (2005). DNA polymerase kappa is specifically required for recovery from the benzo[a]pyrene-dihydrodiol epoxide (BPDE)-induced S-phase checkpoint. J. Biol. Chem..

[109] Ohashi E., Bebenek K., Matsuda T., Feaver W.J., Gerlach V.L., Friedberg E.C., Ohmori H., Kunkel T.A. (2000). Fidelity and processivity of DNA synthesis by DNA polymerase κ, the product of the human DINB1 gene. J. Biol. Chem..

[110] Bavoux C., Leopoldino A.M., Bergoglio V.J.O.W., Ogi T., Bieth A., Judde J.G., Pena S.D., Poupon M.F., Helleday T. (2005). Up-regulation of the error-prone DNA polymerase κ promotes pleiotropic genetic alterations and tumorigenesis. Cancer Res..

[111] Doles J., Oliver T.G., Cameron E.R., Hsu G., Jacks T., Walker G.C., Hemann M.T. (2010). Suppression of Rev3, the catalytic subunit of Polζ, sensitizes drug-resistant lung tumors to chemotherapy. Proc. Natl. Acad. Sci. USA.

[112] Nelson J.R., Lawrence C.W., Hinkle D.C. (1996). Deoxycytidyl transferase activity of yeast REV1 protein. Nature.

[113] Zhang Y., Wu X., Rechkoblit O., Geacintov N.E., Taylor J.S., Wang Z. (2002). Response of human REV1 to different DNA damage: Preferential dCMP insertion opposite the lesion. Nucleic Acids Res..

[114] Nair D.T., Johnson R.E., Prakash L., Prakash S., Aggarwal A.K. (2005). Rev1 employs a novel mechanism of DNA synthesis using a protein template. Science.

[115] Ross A.L., Simpson L.J., Sale J.E. (2005). Vertebrate DNA damage tolerance requires the C-terminus but not BRCT or transferase domains of REV1. Nucleic Acids Res..

[116] Guo C., Fischhaber P.L., Luk-Paszyc M.J., Masuda Y., Zhou J., Kamiya K., Kisker C., Friedberg E.C. (2003). Mouse Rev1 protein interacts with multiple DNA polymerases involved in translesion DNA synthesis. EMBO J..

[117] Ohashi E., Murakumo Y., Kanjo N., Akagi J., Masutani C., Hanaoka F., Ohmori H. (2004). Interaction of hREV1 with three human Y-family DNA polymerases. Genes Cells.

[118] Lee Y.S., Gregory M.T., Yang W. (2014). Human Pol ζ purified with accessory subunits is active in translesion DNA synthesis and complements Pol η in cisplatin bypass. Proc. Natl. Acad. Sci. USA.

[119] Shachar S., Ziv O., Avkin S., Adar S., Wittschieben J., Reissner T., Chaney S., Friedberg E.C., Wang Z., Carell T. (2009). Two-polymerase mechanisms dictate error-free and error-prone translesion DNA synthesis in mammals. EMBO J..

[120] Makarova A.V., Stodola J.L., Burgers P.M. (2012). A four-subunit DNA polymerase ζ complex containing Pol δ accessory subunits is essential for PCNA-mediated mutagenesis. Nucleic Acids Res..

[121] Friedberg E.C., Fischhaber P.L., Kisker C. (2001). Error-prone DNA polymerases: Novel structures and the benefits of infidelity. Cell.

[122] Diaz M., Watson N.B., Turkington G., Verkoczy L.K., Klinman N.R., McGregor W.G. (2003). Decreased frequency and highly aberrant spectrum of ultraviolet-induced mutations in the hprt gene of mouse fibroblasts expressing antisense RNA to DNA polymerase zeta. Mol. Cancer Res..

[123] Despras E., Sittewelle M., Pouvelle C., Delrieu N., Cordonnier A.M., Kannouche P.L. (2016). Rad18-dependent SUMOylation of human specialized DNA polymerase eta is required to prevent under-replicated DNA. Nat. Commun..

[124] Rey L., Sidorova J.M., Puget N., Boudsocq F., Biard D.S., Monnat R.J., Cazaux C., Hoffmann J.S. (2009). Human DNA polymerase eta is required for common fragile site stability during unperturbed DNA replication. Mol. Cell. Biol..

[125] Bergoglio V., Boyer A.S., Walsh E., Naim V., Legube G., Lee M.Y., Rey L., Rosselli F., Cazaux C., Eckert K.A. (2013). DNA synthesis by Pol eta promotes fragile site stability by preventing under-replicated DNA in mitosis. J. Cell Biol..

[126] Buisson R., Niraj J., Pauty J., Maity R., Zhao W., Coulombe Y., Sung P., Masson J.Y. (2014). Breast cancer proteins PALB2 and BRCA2 stimulate polymerase eta in recombination-associated DNA synthesis at blocked replication forks. Cell Rep..

[127] Baptiste B.A., Eckert K.A. (2012). DNA polymerase kappa microsatellite synthesis: Two distinct mechanisms of slippage-mediated errors. Environ. Mol. Mutagen..

[128] Tubbs A., Sridharan S., van Wietmarschen N., Maman Y., Callen E., Stanlie A., Wu W., Wu X., Day A., Wong N. (2018). Dual Roles of Poly(dA:dT) Tracts in Replication Initiation and Fork Collapse. Cell.

[129] Mansilla S.F., Bertolin A.P., Bergoglio V., Pillaire M.J., Gonzalez Besteiro M.A., Luzzani C., Miriuka S.G., Cazaux C., Hoffmann J.S., Gottifredi V. (2016). Cyclin Kinase-independent role of p21(CDKN1A) in the promotion of nascent DNA elongation in unstressed cells. Elife.

[130] Northam M.R., Robinson H.A., Kochenova O.V., Shcherbakova P.V. (2010). Participation of DNA polymerase zeta in replication of undamaged DNA in Saccharomyces cerevisiae. Genetics.

[131] Northam M.R., Moore E.A., Mertz T.M., Binz S.K., Stith C.M., Stepchenkova E.I., Wendt K.L., Burgers P.M., Shcherbakova P.V. (2014). DNA polymerases ζ and Rev1 mediate error-prone bypass of non-B DNA structures. Nucleic Acids Res..

[132] Bhat A., Andersen P.L., Qin Z., Xiao W. (2013). Rev3, the catalytic subunit of Polzeta, is required for maintaining fragile site stability in human cells. Nucleic Acids Res..

[133] Wickramasinghe C.M., Arzouk H., Frey A., Maiter A., Sale J.E. (2015). Contributions of the specialised DNA polymerases to replication of structured DNA. DNA Repair (Amst).

[134] Sarkies P., Reams C., Simpson L.J., Sale J.E. (2010). Epigenetic instability due to defective replication of structured DNA. Mol. Cell.

[135] Wu C.G., Spies M. (2016). G-quadruplex recognition and remodeling by the FANCJ helicase. Nucleic Acids Res..

[136] Sarkies P., Murat P., Phillips L.G., Patel K.J., Balasubramanian S., Sale J.E. (2012). FANCJ coordinates two pathways that maintain epigenetic stability at G-quadruplex DNA. Nucleic Acids Res..

[137] Eddy S., Maddukuri L., Ketkar A., Zafar M.K., Henninger E.E., Pursell Z.F., Eoff R.L. (2015). Evidence for the kinetic partitioning of polymerase activity on G-quadruplex DNA. Biochemistry.

[138] Eddy S., Ketkar A., Zafar M.K., Maddukuri L., Choi J.Y., Eoff R.L. (2014). Human Rev1 polymerase disrupts G-quadruplex DNA. Nucleic Acids Res..

[139] Eddy S., Tillman M., Maddukuri L., Ketkar A., Zafar M.K., Eoff R.L. (2016). Human Translesion Polymerase κ Exhibits Enhanced Activity and Reduced Fidelity Two Nucleotides from G-Quadruplex DNA. Biochemistry.

[140] Betous R., Rey L., Wang G., Pillaire M.J., Puget N., Selves J., Biard D.S., Shin-ya K., Vasquez K.M., Cazaux C., Hoffmann J.S. (2009). Role of TLS DNA polymerases eta and kappa in processing naturally occurring structured DNA in human cells. Mol. Carcinog..

[141] Garcia-Exposito L., Bournique E., Bergoglio V., Bose A., Barroso-Gonzalez J., Zhang S., Roncaioli J.L., Lee M., Wallace C.T., Watkins S.C. (2016). Proteomic Profiling Reveals a Specific Role for Translesion DNA Polymerase η in the Alternative Lengthening of Telomeres. Cell Rep..

[142] Falabella M., Fernandez R.J., Johnson B., Kaufman B.A. (2018). Potential roles for G-quadruplexes in mitochondria. Curr. Med. Chem..

[143] Damas J., Carneiro J., Goncalves J., Stewart J.B., Samuels D.C., Amorim A., Pereira F. (2012). Mitochondrial DNA deletions are associated with non-B DNA conformations. Nucleic Acids Res..

[144] Dong D.W., Pereira F., Barrett S.P., Kolesar J.E., Cao K., Damas J., Yatsunyk L.A., Johnson F.B., Kaufman B.A. (2014). Association of G-quadruplex forming sequences with human mtDNA deletion breakpoints. BMC Genom..

[145] Bharti S.K., Sommers J.A., Zhou J., Kaplan D.L., Spelbrink J.N., Mergny J.L., Brosh R.M. (2014). DNA sequences proximal to human mitochondrial DNA deletion breakpoints prevalent in human disease form G-quadruplexes, a class of DNA structures inefficiently unwound by the mitochondrial replicative Twinkle helicase. J. Biol. Chem..

[146] Yan S., Michael W.M. (2009). TopBP1 and DNA polymerase-α directly recruit the 9-1-1 complex to stalled DNA replication forks. J. Cell Biol..

[147] Bétous R., Pillaire M.J., Pierini L., van der Laan S., Recolin B., Ohl-Séguy E., Guo C., Niimi N., Grúz P., Nohmi T. (2013). DNA polymerase κ-dependent DNA synthesis at stalled replication forks is important for CHK1 activation. EMBO J..

[148] DeStephanis D., McLeod M., Yan S. (2015). REV1 is important for the ATR-Chk1 DNA damage response pathway in Xenopus egg extracts. Biochem. Biophys. Res. Commun..

[149] McIntosh D., Blow J.J. (2012). Dormant origins, the licensing checkpoint, and the response to replicative stresses. Cold Spring Harb. Perspect. Biol..

[150] Futami K., Furuichi Y. (2014). RECQL1 and WRN DNA repair helicases: Potential therapeutic targets and proliferative markers against cancers. Front. Genet..

[151] Buisson R., Niraj J., Rodrigue A., Ho C.K., Kreuzer J., Foo T.K., Hardy E.J., Dellaire G., Haas W., Xia B. (2017). Coupling of Homologous Recombination and the Checkpoint by ATR. Mol. Cell.

[152] Tomida J., Itaya A., Shigechi T., Unno J., Uchida E., Ikura M., Masuda Y., Matsuda S., Adachi J., Kobayashi M. (2013). A novel interplay between the Fanconi anemia core complex and ATR-ATRIP kinase during DNA cross-link repair. Nucleic Acids Res..

[153] Nepal M., Che R., Zhang J., Ma C., Fei P. (2017). Fanconi Anemia Signaling and Cancer. Trends Cancer.

[154] Göhler T., Sabbioneda S., Green C.M., Lehmann A.R. (2011). ATR-mediated phosphorylation of DNA polymerase η is needed for efficient recovery from UV damage. J. Cell Biol..

[155] Bi X., Barkley L.R., Slater D.M., Tateishi S., Yamaizumi M., Ohmori H., Vaziri C. (2006). Rad18 regulates DNA polymerase kappa and is required for recovery from S-phase checkpoint-mediated arrest. Mol. Cell. Biol..

[156] Casper A.M., Nghiem P., Arlt M.F., Glover T.W. (2002). ATR regulates fragile site stability. Cell.

[157] Chen Y.W., Cleaver J.E., Hatahet Z., Honkanen R.E., Chang J.Y., Yen Y., Chou K.M. (2008). Human DNA polymerase eta activity and translocation is regulated by phosphorylation. Proc. Natl. Acad. Sci. USA.

[158] Zannini L., Delia D., Buscemi G. (2014). CHK2 kinase in the DNA damage response and beyond. J. Mol. Cell. Biol..

[159] Di Micco R., Fumagalli M., Cicalese A., Piccinin S., Gasparini P., Luise C., Schurra C., Garre M., Nuciforo P.G., Bensimon A. (2006). Oncogene-induced senescence is a DNA damage response triggered by DNA hyper-replication. Nature.

[160] Bartkova J., Rezaei N., Liontos M., Karakaidos P., Kletsas D., Issaeva N., Vassiliou L.V., Kolettas E., Niforou K., Zoumpourlis V.C. (2006). Oncogene-induced senescence is part of the tumorigenesis barrier imposed by DNA damage checkpoints. Nature.

[161] Miron K., Golan-Lev T., Dvir R., Ben-David E., Kerem B. (2015). Oncogenes create a unique landscape of fragile sites. Nat. Commun..

[162] Croce C.M. (2008). Oncogenes and cancer. N. Engl. J. Med..

[163] Shortt J., Johnstone R.W. (2012). Oncogenes in cell survival and cell death. Cold Spring Harb. Perspect. Biol..

[164] Dereli-Öz A., Versini G., Halazonetis T.D. (2011). Studies of genomic copy number changes in human cancers reveal signatures of DNA replication stress. Mol. Oncol..

[165] Gorgoulis V.G., Vassiliou L.V., Karakaidos P., Zacharatos P., Kotsinas A., Liloglou T., Venere M., Ditullio R.A., Kastrinakis N.G., Levy B. (2005). Activation of the DNA damage checkpoint and genomic instability in human precancerous lesions. Nature.

[166] Herold S., Herkert B., Eilers M. (2009). Facilitating replication under stress: An oncogenic function of MYC?. Nat. Rev. Cancer.

[167] Neelsen K.J., Zanini I.M., Herrador R., Lopes M. (2013). Oncogenes induce genotoxic stress by mitotic processing of unusual replication intermediates. J. Cell Biol..

[168] Sanjiv K., Hagenkort A., Calderón-Montaño J.M., Koolmeister T., Reaper P.M., Mortusewicz O., Jacques S.A., Kuiper R.V., Schultz N., Scobie M. (2016). Cancer-Specific Synthetic Lethality between ATR and CHK1 Kinase Activities. Cell Rep..

[169] Karnitz L.M., Zou L. (2015). Molecular Pathways: Targeting ATR in Cancer Therapy. Clin. Cancer Res..

[170] Kotsantis P., Petermann E., Boulton S.J. (2018). Mechanisms of Oncogene-Induced Replication Stress: Jigsaw Falling into Place. Cancer Discov..

[171] Pylayeva-Gupta Y., Grabocka E., Bar-Sagi D. (2011). RAS oncogenes: Weaving a tumorigenic web. Nat. Rev. Cancer.

[172] Fang X., Yu S., Eder A., Mao M., Bast R.C., Boyd D., Mills G.B. (1999). Regulation of BAD phosphorylation at serine 112 by the Ras-mitogen-activated protein kinase pathway. Oncogene.

[173] Rosen K., Rak J., Jin J., Kerbel R.S., Newman M.J., Filmus J. (1998). Downregulation of the pro-apoptotic protein Bak is required for the ras-induced transformation of intestinal epithelial cells. Curr. Biol..

[174] Zhao J., Kennedy B.K., Lawrence B.D., Barbie D.A., Matera A.G., Fletcher J.A., Harlow E. (2000). NPAT links cyclin E-Cdk2 to the regulation of replication-dependent histone gene transcription. Genes Dev..

[175] Cooley A., Zelivianski S., Jeruss J.S. (2010). Impact of cyclin E overexpression on Smad3 activity in breast cancer cell lines. Cell Cycle.

[176] Conacci-Sorrell M., McFerrin L., Eisenman R.N. (2014). An overview of MYC and its interactome. Cold Spring Harb. Perspect. Med..

[177] Chappell J., Dalton S. (2013). Roles for MYC in the establishment and maintenance of pluripotency. Cold Spring Harb. Perspect. Med..

[178] Neiman P.E., Thomas S.J., Loring G. (1991). Induction of apoptosis during normal and neoplastic B-cell development in the bursa of Fabricius. Proc. Natl. Acad. Sci. USA.

[179] Hernandez-Segura A., Nehme J., Demaria M. (2018). Hallmarks of Cellular Senescence. Trends Cell Biol..

[180] Alevizopoulos K., Vlach J., Hennecke S., Amati B. (1997). Cyclin E and c-Myc promote cell proliferation in the presence of p16INK4a and of hypophosphorylated retinoblastoma family proteins. EMBO J..

[181] Chakradeo S., Elmore L.W., Gewirtz D.A. (2016). Is Senescence Reversible?. Curr. Drug Targets.

[182] Hydbring P., Larsson L.G. (2010). Cdk2: A key regulator of the senescence control function of Myc. Aging (Albany NY).

[183] Fragkos M., Ganier O., Coulombe P., Méchali M. (2015). DNA replication origin activation in space and time. Nat. Rev. Mol. Cell Biol..

[184] Langston L.D., Mayle R., Schauer G.D., Yurieva O., Zhang D., Yao N.Y., Georgescu R.E., O’Donnell M.E. (2017). Mcm10 promotes rapid isomerization of CMG-DNA for replisome bypass of lagging strand DNA blocks. Elife.

[185] Ricke R.M., Bielinsky A.K. (2004). Mcm10 regulates the stability and chromatin association of DNA polymerase-alpha. Mol. Cell.

[186] Sawyer S.L., Cheng I.H., Chai W., Tye B.K. (2004). Mcm10 and Cdc45 cooperate in origin activation in Saccharomyces cerevisiae. J. Mol. Biol..

[187] Langston L.D., Zhang D., Yurieva O., Georgescu R.E., Finkelstein J., Yao N.Y., Indiani C., O’Donnell M.E. (2014). CMG helicase and DNA polymerase epsilon form a functional 15-subunit holoenzyme for eukaryotic leading-strand DNA replication. Proc. Natl. Acad. Sci. USA.

[188] Sanada I., Nakada K., Furugen S., Kumagai E., Yamaguchi K., Yoshida M., Takatsuki K. (1986). Chromosomal abnormalities in a patient with smoldering adult T-cell leukemia: Evidence for a multistep pathogenesis. Leuk. Res..

[189] Borlado L.R., Mendez J. (2008). CDC6: From DNA replication to cell cycle checkpoints and oncogenesis. Carcinogenesis.

[190] Tatsumi Y., Sugimoto N., Yugawa T., Narisawa-Saito M., Kiyono T., Fujita M. (2006). Deregulation of Cdt1 induces chromosomal damage without rereplication and leads to chromosomal instability. J. Cell Sci..

[191] Hills S.A., Diffley J.F. (2014). DNA replication and oncogene-induced replicative stress. Curr. Biol..

[192] Zheng D., Ye S., Wang X., Zhang Y., Yan D., Cai X., Gao W., Shan H., Gao Y., Chen J. (2017). Pre-RC Protein MCM7 depletion promotes mitotic exit by Inhibiting CDK1 activity. Sci. Rep..

[193] Ekholm-Reed S., Méndez J., Tedesco D., Zetterberg A., Stillman B., Reed S.I. (2004). Deregulation of cyclin E in human cells interferes with prereplication complex assembly. J. Cell Biol..

[194] Jones R.M., Mortusewicz O., Afzal I., Lorvellec M., García P., Helleday T., Petermann E. (2013). Increased replication initiation and conflicts with transcription underlie Cyclin E-induced replication stress. Oncogene.

[195] Dominguez-Sola D., Ying C.Y., Grandori C., Ruggiero L., Chen B., Li M., Galloway D.A., Gu W., Gautier J., Dalla-Favera R. (2007). Non-transcriptional control of DNA replication by c-Myc. Nature.

[196] Valovka T., Schönfeld M., Raffeiner P., Breuker K., Dunzendorfer-Matt T., Hartl M., Bister K. (2013). Transcriptional control of DNA replication licensing by Myc. Sci. Rep..

[197] Murga M., Campaner S., Lopez-Contreras A.J., Toledo L.I., Soria R., Montaña M.F., Artista L., Schleker T., Guerra C., Garcia E. (2011). Exploiting oncogene-induced replicative stress for the selective killing of Myc-driven tumors. Nat. Struct. Mol. Biol..

[198] Wang W.J., Wu S.P., Liu J.B., Shi Y.S., Huang X., Zhang Q.B., Yao K.T. (2013). MYC regulation of CHK1 and CHK2 promotes radioresistance in a stem cell-like population of nasopharyngeal carcinoma cells. Cancer Res..

[199] Moser R., Toyoshima M., Robinson K., Gurley K.E., Howie H.L., Davison J., Morgan M., Kemp C.J., Grandori C. (2012). MYC-driven tumorigenesis is inhibited by WRN syndrome gene deficiency. Mol. Cancer Res..

[200] Hall J.R., Lee H.O., Bunker B.D., Dorn E.S., Rogers G.C., Duronio R.J., Cook J.G. (2008). Cdt1 and Cdc6 are destabilized by rereplication-induced DNA damage. J. Biol. Chem..

[201] Davidson I.F., Li A., Blow J.J. (2006). Deregulated replication licensing causes DNA fragmentation consistent with head-to-tail fork collision. Mol. Cell.

[202] Ho A., Dowdy S.F. (2002). Regulation of G(1) cell-cycle progression by oncogenes and tumor suppressor genes. Curr. Opin. Genet. Dev..

[203] Macheret M., Halazonetis T.D. (2018). Intragenic origins due to short G1 phases underlie oncogene-induced DNA replication stress. Nature.

[204] Kimmelman A.C. (2015). Metabolic Dependencies in RAS-Driven Cancers. Clin. Cancer Res..

[205] Guo J.Y., Chen H.Y., Mathew R., Fan J., Strohecker A.M., Karsli-Uzunbas G., Kamphorst J.J., Chen G., Lemons J.M., Karantza V. (2011). Activated Ras requires autophagy to maintain oxidative metabolism and tumorigenesis. Genes Dev..

[206] Lim J.H., Lee E.S., You H.J., Lee J.W., Park J.W., Chun Y.S. (2004). Ras-dependent induction of HIF-1α785 via the Raf/MEK/ERK pathway: A novel mechanism of Ras-mediated tumor promotion. Oncogene.

[207] Liu Y.C., Li F., Handler J., Huang C.R., Xiang Y., Neretti N., Sedivy J.M., Zeller K.I., Dang C.V. (2008). Global regulation of nucleotide biosynthetic genes by c-Myc. PLoS ONE.

[208] Li F., Wang Y., Zeller K.I., Potter J.J., Wonsey D.R., O’Donnell K.A., Kim J.W., Yustein J.T., Lee L.A., Dang C.V. (2005). Myc stimulates nuclearly encoded mitochondrial genes and mitochondrial biogenesis. Mol. Cell. Biol..

[209] Kim J.W., Zeller K.I., Wang Y., Jegga A.G., Aronow B.J., O’Donnell K.A., Dang C.V. (2004). Evaluation of myc E-box phylogenetic footprints in glycolytic genes by chromatin immunoprecipitation assays. Mol. Cell. Biol..

[210] Wise D.R., DeBerardinis R.J., Mancuso A., Sayed N., Zhang X.Y., Pfeiffer H.K., Nissim I., Daikhin E., Yudkoff M., McMahon S.B. (2008). Myc regulates a transcriptional program that stimulates mitochondrial glutaminolysis and leads to glutamine addiction. Proc. Natl. Acad. Sci. USA.

[211] Kelekar A., Cole M.D. (1987). Immortalization by c-myc, H-ras, and Ela oncogenes induces differential cellular gene expression and growth factor responses. Mol. Cell. Biol..

[212] Elenbaas B., Spirio L., Koerner F., Fleming M.D., Zimonjic D.B., Donaher J.L., Popescu N.C., Hahn W.C., Weinberg R.A. (2001). Human breast cancer cells generated by oncogenic transformation of primary mammary epithelial cells. Genes Dev..

[213] Aird K.M., Worth A.J., Snyder N.W., Lee J.V., Sivanand S., Liu Q., Blair I.A., Wellen K.E., Zhang R. (2015). ATM couples replication stress and metabolic reprogramming during cellular senescence. Cell Rep..

[214] Maya-Mendoza A., Ostrakova J., Kosar M., Hall A., Duskova P., Mistrik M., Merchut-Maya J.M., Hodny Z., Bartkova J., Christensen C. (2015). Myc and Ras oncogenes engage different energy metabolism programs and evoke distinct patterns of oxidative and DNA replication stress. Mol. Oncol..

[215] Yang Y., Gao Y., Mutter-Rottmayer L., Zlatanou A., Durando M., Ding W., Wyatt D., Ramsden D., Tanoue Y., Tateishi S. (2017). DNA repair factor RAD18 and DNA polymerase Polκ confer tolerance of oncogenic DNA replication stress. J. Cell Biol..

[216] Mertz T.M., Sharma S., Chabes A., Shcherbakova P.V. (2015). Colon cancer-associated mutator DNA polymerase δ variant causes expansion of dNTP pools increasing its own infidelity. Proc. Natl. Acad. Sci. USA.

[217] Williams L.N., Marjavaara L., Knowels G.M., Schultz E.M., Fox E.J., Chabes A., Herr A.J. (2015). dNTP pool levels modulate mutator phenotypes of error-prone DNA polymerase ε variants. Proc. Natl. Acad. Sci. USA.

[218] Mathews C.K. (2015). Deoxyribonucleotide metabolism, mutagenesis and cancer. Nat. Rev. Cancer.

[219] Aird K.M., Zhang G., Li H., Tu Z., Bitler B.G., Garipov A., Wu H., Wei Z., Wagner S.N., Herlyn M. (2013). Suppression of nucleotide metabolism underlies the establishment and maintenance of oncogene-induced senescence. Cell Rep..

[220] Mannava S., Moparthy K.C., Wheeler L.J., Natarajan V., Zucker S.N., Fink E.E., Im M., Flanagan S., Burhans W.C., Zeitouni N.C. (2013). Depletion of deoxyribonucleotide pools is an endogenous source of DNA damage in cells undergoing oncogene-induced senescence. Am. J. Pathol..

[221] Schmidt T.T., Reyes G., Gries K., Ceylan C., Sharma S., Meurer M., Knop M., Chabes A., Hombauer H. (2017). Alterations in cellular metabolism triggered by. Proc. Natl. Acad. Sci. USA.

[222] Bester A.C., Roniger M., Oren Y.S., Im M.M., Sarni D., Chaoat M., Bensimon A., Zamir G., Shewach D.S., Kerem B. (2011). Nucleotide deficiency promotes genomic instability in early stages of cancer development. Cell.

[223] Liu X., Disbrow G.L., Yuan H., Tomaic V., Schlegel R. (2007). Myc and human papillomavirus type 16 E7 genes cooperate to immortalize human keratinocytes. J. Virol..

[224] Kurashima K., Sekimoto T., Oda T., Kawabata T., Hanaoka F., Yamashita T. (2018). Polη, a Y-family translesion synthesis polymerase, promotes cellular tolerance of Myc-induced replication stress. J. Cell Sci..

[225] Hedglin M., Pandey B., Benkovic S.J. (2016). Characterization of human translesion DNA synthesis across a UV-induced DNA lesion. Elife.

[226] Wittig B., Wölfl S., Dorbic T., Vahrson W., Rich A. (1992). Transcription of human c-myc in permeabilized nuclei is associated with formation of Z-DNA in three discrete regions of the gene. EMBO J..

[227] Belotserkovskii B.P., De Silva E., Tornaletti S., Wang G., Vasquez K.M., Hanawalt P.C. (2007). A triplex-forming sequence from the human c-MYC promoter interferes with DNA transcription. J. Biol. Chem..

[228] Siddiqui-Jain A., Grand C.L., Bearss D.J., Hurley L.H. (2002). Direct evidence for a G-quadruplex in a promoter region and its targeting with a small molecule to repress c-MYC transcription. Proc. Natl. Acad. Sci. USA.

[229] Del Mundo I.M.A., Zewail-Foote M., Kerwin S.M., Vasquez K.M. (2017). Alternative DNA structure formation in the mutagenic human c-MYC promoter. Nucleic Acids Res..

[230] Barnes R.P., Tsao W., Moldovan G., Eckert K. (2018). DNA Polymerase Eta Prevents Tumor Cell Cycle Arrest and Cell Death During Recovery from Replication Stress. Cancer Res..

[231] Desai A., Yan Y., Gerson S.L. (2018). Advances in therapeutic targeting of the DNA damage response in cancer. DNA Repair (Amst).

